# Validation of Multi-Residue GC-MS/MS and LC-MS/MS Methods for the Analysis of Pesticide Residues in Botanicals

**DOI:** 10.3390/foods15213892

**Published:** 2026-10-30

**Authors:** Elena Butovskaya, Giuseppina Castrezzati, Giampiero Migliorato, Gregorio Peron, Mara Gasparini

**Affiliations:** 1 Food and Feed Chemistry Department, Istituto Zooprofilattico Sperimentale della Lombardia e dell’Emilia Romagna “Bruno Ubertini” (IZSLER), 25124 Brescia, Italy; giuseppina.castrezzati@izsler.it (G.C.); mara.gasparini@izsler.it (M.G.); 2 Department of Molecular and Translational Medicine, University of Brescia, 25123 Brescia, Italy; gregorio.peron@unibs.it

**Keywords:** pesticide residues, multi-residue analytical methods, liquid chromatography–tandem mass spectrometry, gas chromatography–tandem mass spectrometry, validation, botanicals food supplements

## Abstract

Botanical food supplements are increasingly consumed worldwide. As finished products, they may be contaminated with pesticide residues, which can potentially become concentrated relative to the raw botanical materials during processing steps. In this study, analytical methods were developed for the quantification of 252 pesticide residues in botanical food supplements using LC-MS/MS and GC-MS/MS, with both techniques employing a common sample preparation procedure. Sample extraction and clean-up were performed using a QuEChERS-based approach combined with dispersive solid-phase clean-up step using graphitized carbon black (GCB) and primary–secondary amine (PSA) sorbents followed by Oasis^®^ WAX cartridge purification. Method validation was performed in accordance with the EU guidance document SANTE/11312/2021. The validated methods were subsequently applied to ten plant extract samples to assess their applicability to different botanical food supplement matrices. Multiple pesticide residues were detected in three samples, with concentrations ranging from 0.01 to 0.19 mg/kg. The application of the methods to real samples confirmed suitability for the analysis of diverse botanical matrices and underscored the need for robust analytical approaches to support the routine monitoring of pesticide residues in botanical food supplements and ensure consumer safety.

## 1. Introduction

The global consumption of food supplements has grown steadily in recent years, accompanied by a continuous expansion of the market and product availability [1,2,3]. The main factors driving this growth include population aging, increased awareness of preventive healthcare and self-care, and socioeconomic changes that have promoted greater consumer interest in health and wellness [3,4]. Plant-based food supplements, also known as “botanicals,” are manufactured from a wide variety of plant species and plant parts, including leaves, roots, bark, flowers, fruits, seeds, and whole plants. The botanical food supplement market encompasses thousands of species used either individually or in combination in a broad range of formulations. Although these products are often perceived by consumers as being inherently safe because they are of natural origin, several quality and safety concerns have been identified [5,6]. These include variability in the composition of botanical raw materials, adulteration and misidentification of plant species, inconsistencies arising from manufacturing processes and product standardization, as well as contamination with environmental pollutants, mycotoxins, heavy metals, and pesticide residues [7,8,9]. Particularly, although pesticides are widely used to improve agricultural yields and to protect plant materials during storage, transport, and processing from fungal growth and insect infestation, concerns remain regarding their improper use and the occurrence of pesticide residues in plant-derived products. Plant-based food supplements are generally manufactured from dried plant materials obtained from specific plant parts or from more refined extracts produced by solvent extraction of desirable groups of compounds. These extracts are often subsequently standardized to ensure a defined content of specific bioactive compounds or classes of compounds, such as, for instance, polyphenols [10]. Several of these processing steps may also lead to the concentration of pesticide residues, resulting in higher residue levels in the final product than in the original raw plant material [11,12].

In the European Union, maximum residue limits (MRLs) for pesticides in food and feed are established under Regulation (EC) 396/2005 [13]. However, this legislation applies only to raw commodities and no residue limits are available for the final food supplement products (processed and/or composite). According to the same regulation, processing and mixing factors are to be used to derive the corresponding MRLs, which would be applicable to the final product. Nevertheless, the derivation of reliable processing factors remains challenging, as highlighted in the SANTE/10704/2021 guidance document [14].

In Italy, food supplements are not included as a specific commodity group in the national pesticide residue monitoring plan, although they may be investigated through targeted surveillance programs. Consequently, information on pesticide residue occurrence in these products remains limited despite their rapidly increasing consumption.

In recent years, analytical methodologies for the determination of pesticide residues in food have been predominantly based on liquid chromatography (LC) or gas chromatography (GC) coupled with mass spectrometry (MS). These techniques provide the high sensitivity, selectivity, and quantitative accuracy required for the determination of pesticide residues at trace concentration levels. A substantial body of literature is available on the analysis of pesticide residues in plant-derived foods, as reviewed by [15,16,17].

Among various food matrices of plant origin, dietary supplements represent a particularly challenging food category for pesticide residue analysis due to their highly diverse composition. These products vary considerably in their biochemical matrix, water and protein content, and often contain concentrated matrix components, such as pigments, polyphenols, lipids, or other bioactive compounds, which may interfere with analyte extraction and instrumental detection [18,19]. Consequently, sample preparation requires careful optimization, with extraction and clean-up procedures commonly based on approaches such as QuEChERS (Quick, Easy, Cheap, Effective, Rugged, and Safe) methods, solid-phase extraction (SPE), or solid–liquid extraction (SLE) [20,21,22,23,24,25,26,27].

Several analytical methods have been specifically developed for pesticide residue determination in botanical dietary supplements. For example, Paleníková et al. developed a GC–MS/MS method for the quantification of more than 170 pesticide residues in soy-based nutraceuticals, whereas Nieto-García et al. validated an LC–MS/MS method for the determination of 106 pesticide residues in dietary supplements derived from grape seed extracts [28,29]. More recently, Koshy et al. developed a comprehensive multi-residue analytical method for botanical products by combining GC–MS/MS and LC–MS/MS, extending the applicability of pesticide residue analysis across a broad range of botanical matrices [30]. Multi-residue methods were developed for complex food matrices, such as roasted coffee, green tea and curry testing the performance of the method for 172 pesticides [31] and spices, such as cumin and coriander (215 pesticide residues) [32] applying GC-MS/MS detection. Mello et al. validated 65 pesticides in dry herbs used for tea preparation while in a very recent work from Wang et al. an LC-MS/MS method comprising 589 pesticide residues was validated for traditional Chinese medicines [33,34].

The aim of this study was to develop and validate a multi-residue analytical method for the detection and quantification of a broad range of pesticide residues in plant-based food supplements by combining LC–MS/MS and GC–MS/MS analysis with a single sample preparation procedure, thereby extending the range of detectable analytes while maintaining a simple and efficient analytical workflow. The applicability of the method to routine analysis was subsequently assessed by analyzing plant-based botanical samples from local market.

## 2. Materials and Methods

### 2.1. Reagents and Standards

HPLC grade acetonitrile and methanol, LC-MS/MS grade 99% formic acid and acetone were purchased from Carlo Erba Reagents Srl (Cornaredo, MI, Italy), while 10M ammonium formate solution was acquired from Merck (Darmstadt, Germany). For dispersive solid phase extraction (dSPE) Cleanert MAS-Q (Tianjin, China) 15 mL tubes containing 150 mg PSA (primary secondary amine) and 900 mg magnesium sulphate (MgSO_4_) and DisQuE^TM^ 15 mL tubes from Waters (Milford, MA, USA) containing 900 mg MgSO_4_, 450 mg PSA, 300 mg C18 and 50 mg GCB (graphitized carbon black) were tested during the development of the method.

Following custom analytical standard solutions for LC-MS/MS analysis were purchased from CPAchem LTD (Bogomilovo, Bulgary): (i) Aldicarb, Aldicarb-sulfone, Aldicarb-sulfoxide, Carbaryl, Carbofuran, Carbofuran-3-hydroxy, Fenoxycarb, Methiocarb, Methomyl, Oxamyl, Pirimicarb, Pirimicarb-desmethyl, Propoxur, Thiodicarb, Chlorpropham, Diethofencarb 100 mg/L in acetonitrile/acetone 0.01%; (ii) Acephate, Azinphos-ethyl, Azinphos-methyl, Chlorfenvinphos, Chlorpyrifos (Chlorpyrifos-ethyl), Demeton-S-methyl, Demeton-S-methyl-sulfone, Demeton-S-methyl-sulfoxide, Diazinon, Dichlorvos, Dimethoate, Ethion, Ethoprophos, Omethoate, Monocrotophos, Pyrazophos, Triazophos, Fenthion, Fenthion-oxon-sulfone, Fenthion-oxon-sulfoxide, Fenthion-oxon, Fenthion-sulfone, Fenthion-sulfoxide, Malaoxon, Malathion 100 mg/L in acetonitrile/acetone 0.01%; (iii) Fenamiphos, Fenamiphos-sulfone, Fenamiphos-sulfoxide, Fenitrothion, Methamidophos, Metidathion, Paraoxon-ethyl, Paraoxon-methyl, Phosalone, Phosphamidon, Phoxim, Pirimiphos-methyl, Profenofos, Amitraz, N-(2,4-Dimethylphenyl)-N’-methylformamide, N-(2,4-Dimethylphenyl)formamide, Formetanate hydrochloride, Propamocarb hydrochloride, Ethiofencarb, Furathiocarb, Clofentezine, Fenamidone, Dimethomorph, Mandipropamid, Spirodiclofen 100 mg/L in acetonitrile/acetone 0.01%; (iv) Pencycuron, Fenpropathrin, Fenarimol, Bupirimate, Quinoxyfen, Indoxacarb, Metalaxyl-M, Bromuconazole, Bitertanol, Cyproconazole, Difenoconazole, Epoxiconazole, Fenbuconazole, Fluquinconazole, Flusilazole, Flutriafol, Hexaconazole, Metconazole, Myclobutanil, Paclobutrazol, Penconazole, Propiconazole, Prothioconazole-desthio, Dodine, Fosthiazate 100 mg/L in acetonitrile/acetone (1/1); (v) Tebuconazole, Tetraconazole, Triadimefon, Triadimenol, Triticonazole, Linuron, Azoxystrobin, Pyraclostrobin, Kresoxim-methyl, Trifloxystrobin, Acetamiprid, Clothianidin, Imidacloprid, Thiacloprid, Thiamethoxam, Spinosad, Hexythiazox, Iprovalicarb, Lufenuron, Methoxyfenozide, Zoxamide, Flufenoxuron, Triflumuron, Boscalid, Tolclofos-methyl 100 mg/L in acetonitrile/acetone 0.01%; (vi) Bixafen, Chlorantraniliprole, Cyazofamid, Cyprosulfamide, Diniconazole, Ethirimol, Famoxadone, Fenpropidin, Fensulfothion, Flufenacet, Fluopicolide, Iodosulfuron-methyl-sodium, Isoprothiolane, Isoxaflutole, Mefenpyr-diethyl, Mesosulfuron-methyl, Metrafenone, Metribuzin, Propham, Pymetrozine, Terbuthylazine, Thiencarbazone-methyl, Parathion 100 mg/L in acetonitrile; (vii) Cyhalofop-butyl, Cymoxanil, Cyromazine, DMST, Fenpyroximate, Flonicamid, Flubendiamide, Fluopyram, Propaquizafop, Quizalofop-P-ethyl, Tolylfluanid 100 mg/L in acetonitrile; (viii) Tebufenozide, Fenhexamid, Imazalil, Prochloraz, Fenpropimorph, Spiroxamine, Etofenprox, Propargite, Pyrimethanil, Thiabendazole, Thiophanate-methyl in 100 mg/L in acetonitrile/acetone 0.01%; (ix) Ametoctradin, Chlorbufam, Cyantralinprole, Cyflufenamid, Diflubenzuron, Diflufenican, Dinotefuran, Fenpyrazamine, Fluxapyroxad, Metaflumizone, Proquinazid, Prosulfocarb, Prothiophos, Pyridalyl, Quinalphos, Spinetoram, Spirotetramat, Sulfoxaflor, Tolfenpyrad, Tricyclazole, Triforine, Oxyfluorfen 100 mg/L in acetonitrile, (x) Carbendazim 100 mg/L in acetonitrile. Custom standard solution containing Flupyradifurone, Metobromuron, Metazachlor, BTS 44595, Prochloraz metabolite BTS 44596, Flutolanil, Penflufen, Isofetamid, Triflumizole, Penthiopyrad, Triflumizole-amino, Quinoclamine, Metamitron, Oxadiargyl, Mefentrifluconazole, Benzovindiflupyr 100 mg/L in acetonitrile was purchased from Dr Ehrenstorfer GmBH (Augsburg, Germany). Single internal standard (IS) solutions at 100 mg/L in acetone were purchased from Dr Ehrenstorfer GmBH (Augsburg, Germany): Amitraz-D12, Azinphos-ethyl-D10, Dimethoate-D6, Etphenprox-D5, Propiconazole-D5. Carbendazim-D3, Cyromazine-D4 and Imidacloprid-D4 were purchased from Dr Ehrenstorfer GmBH (Augsburg, Germany) in powder form.

For GC-MS/MS analysis custom analytical standard solutions at the concentration of 250 mg/L in acetone were also obtained from CPAchem LTD (Bogomilovo, Bulgary): (i) Bifenthrin, Chlorpyrifos methyl, Cyfluthrin, lambda-Cyhalotrin, Cypermethrin, Deltamethrin, Endosulfan-total (sulfate), Fenvalerate, tau-Fluvalinate, Paraoxon-methyl, Parathion-methyl, Pendimethalin, Permethrin, Phosmet, Malathion, 2,4′-DDD, 2,4′-DDE, Vinclozolin; (ii) Acrinathrin, Azinphos-methyl, Bromopropylate, Buprofezin, Cadusafos, Coumaphos, Cyprodinil, Dicofol, Dicloran, Diphenylamine, Endosulfan-alpha, Endosulfan-beta, Fenazaquin, Fenitrothion, Fenpropathrin, Fipronil, Fludioxonil, Alfa-HCH, Beta-HCH, Iprodione, gamma-HCH (Lindane), Mepanipyrim, Methacrifos, Oxadixyl, Phenthoate; (iii) Procymidone, Propyzamide, Pyridaben, Pyriproxyfen, Tebyfenpyrad, Tefluthrin, Tetradifon, Trichlorfon, Trifluralin, 2-Phenylphenol, Chlorfenapyr, EPN (Ethyl p-nitrophenyl benzenethiophosphonate), Isofenphos-methyl, 2,4′-DDT, 4,4′-DDD (TDE), 4,4′-DDE, 4,4′-DDT; (iv) Aldrin, Dieldrin, Isocarbofos, Heptachlor, Heptachlor-endo-epoxide, Heptachlor-exo-epoxide, Endrin, Hexachlorobenzene, Nitrofen, Spiromesifen, Propham, Chlorpropham, Terbufos, Disulfoton, Disulfoton-sulfone, Disulfoton-sulfoxide, Parathion, cis-Chlordane, trans-Chlordane, oxy-Chlordane, Methoxychlor (DMTD), Meviphos; (v) Etofenprox, Etoxazole, Fipronil-sulfone, Pirimiphos-ethyl, Tetramethrin. Aclonifen, Chlorobenzilate and Quintozene were purchased from Dr Ehrenstorfer GmBH (Augsburg, Germany) in powder form. Single IS solutions at 100 mg/L in acetone were purchased from Dr Ehrenstorfer GmBH (Augsburg, Germany): 2,4′-DDT-D8, 4,4′-DDE-D8, 4,4′-DDT-D8, alpha-HCH-D6, Hexachlorobenzene-13C6, while Lindane-D6 was in cyclohexane. Chlorpyrifos-methyl-D6 was purchased from Dr Ehrenstorfer GmBH (Augsburg, Germany) in powder form.

### 2.2. Pool Standard Solutions Preparation

An intermediate pool standard solution for target analytes at 5 µg/mL and 10 µg/mL in acetonitrile were prepared for LC-MS/MS and GC-MS/MS analysis, respectively, starting from custom standard solutions. The intermediate standard solutions were diluted to obtain the working solutions at 1 µg/mL. LC-MS/MS IS working solution was prepared at 50 µg/mL for Cyromazine-D4 and 10 µg/mL for all other ISs in acetonitrile and then diluted to IS working solution at 5–1 µg/mL. GC-MS/MS IS intermediate solution was prepared at 3 µg/mL for 4,4′-DDE-D8 and 10 µg/mL for all other ISs and then diluted to a working IS solution 0.3–1 µg/mL. Dimethoate-D6 syringe standard solution for the control of LC-MS/MS sample injection was prepared in water at 0.005 µg/mL starting from the diluted standard solution in acetone at 0.4 µg/mL. Lindane-D6 syringe standard solution for the control of GC-MS/MS sample injection was prepared in ethyl acetate at the concentration of 0.125 µg/mL starting from the diluted standard solution at 10 µg/mL.

### 2.3. Sample Preparation

**Figure 1:**
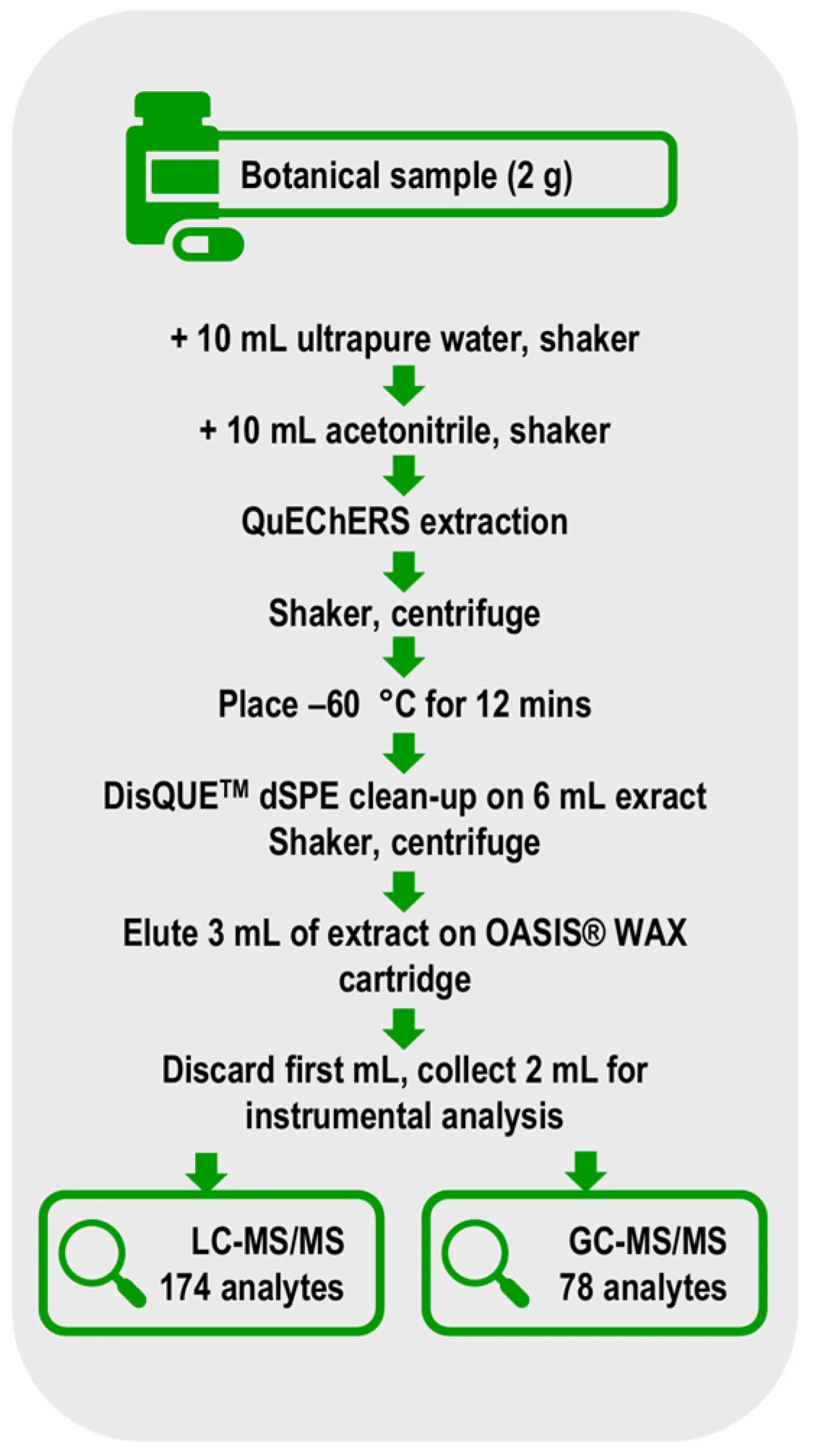
Schematic representation of botanical sample preparation for LC-MS/MS and GC-MS/MS analysis.

A portion of 2 g of each homogenized sample was weighted in 50 mL polypropylene tube. The LC-MS/MS IS working solution at 50–10 µg/mL was added to reach the final concentration of 50–10 ng/mL and the GC-MS/MS IS working solution at 10–3 µg/mL was added to reach the final concentration of 10–3 ng/mL. Samples were diluted with 10 mL of ultrapure water and shaken on a horizontal shaker for 30 min. Subsequently, 10 mL of acetonitrile was added, and the samples were shaken manually for 1 min. QuEChERS extraction salts (Cleanert, Tianjin, China), containing 4 g magnesium sulphate, 1 g sodium chloride, 1 g sodium citrate, 0.5 g disodium citrate sesquihydrate, were then added to each tube, followed by manual shaking for 1 min and horizontal shaking for additional 3 min. The samples were centrifuged at 4000 rpm for 10 min, after which the upper layer was transferred to a 15 mL polypropylene tube and placed at −60 °C for 12 min. A second centrifugation was performed at subzero temperature (4000 rpm, 10 min), and the cold extract was transferred to a clean polypropylene tube and allowed to equilibrate to room temperature. Dispersive solid-phase extraction (d-SPE) (DisQuE^TM^, Waters, Milford, MA, USA) clean-up was performed on 6 mL of the extract by shaking for 30 s, followed by centrifugation at 4000 rpm for 7 min. Subsequently, 3 mL of the cleaned extract was loaded onto Oasis ^®^ WAX purification cartridge (Waters, Milford, MA, USA) and allowed to percolate under gravity. The first 1 mL of the eluate was discarded, and the remaining 2 mL was collected in a vial for instrumental analysis. The schematic representation of the sample preparation process is shown in Figure 1.

### 2.4. LC-MS/MS Analysis

Samples were injected in a LC-MS/MS platform consisting of HPLC Agilent model 1290 (Agilent Technologies, Santa Clara, CA, USA) coupled with triple quadrupole mass spectrometer Agilent 6470. For chromatographic separation, an Acquity UPLC HSS T3 (Waters, Milford, MA, USA) column (100 mm × 2.1 mm, particle size 1.8 µm) combined with Acquity UPLC HSS T3 (5 mm × 2.1 mm, particle size 1.8 µm) guard column was used as stationary phase. Mobile phase A was an aqueous solution of 2 mM ammonium formate and 2 mM formic acid with the addition of 2% of methanol, while the organic mobile phase B was 2 mM ammonium formate and 2 mM formic acid in methanol. The following binary gradient was used: starting with 95% A hold for 1 min, at 3 min 55% A, reaching 5% A at 16 min and hold until 20.4 min, then reaching the initial conditions at 20.5 min with 4 min of post run equilibration. The chromatographic run was operated at 0.3 mL/min flow rate at the temperature of 40 °C, injecting a total volume of 10 µL of sample in a sandwich mode, starting with 5 µL of syringe standard Dimetoate-D6, 1 µL of sample and final 4 µL of Dimetoate-D6 solution. Mass spectrometer was operated in positive electrospray ionization (ESI+) mode. Following source parameters were set for analysis: capillary voltage 3500 V, gas flow 13 L/min, nebulizer 50 psi, sheath gas flow 12 L/min, gas temperature 120 °C, sheath gas temperature 325 °C, nozzle voltage 300 V. For each analyte, the most abundant MRM (multiple reaction monitoring) transitions, optimized using the reference solutions of the analytes, were monitored. Monitored parent and fragment ions together with the fragmentation parameters are shown in Table S1.

### 2.5. GC-MS/MS Analysis

GC-MS/MS analysis was performed with an Agilent 7890B GC system coupled with an Agilent 7010 triple quadrupole mass spectrometer (Agilent Technologies, Santa Clara, CA, USA). For chromatographic separation, a ZB-MultiResidue-1 (30 m, 0.25 mm ID, 0.25 µm film) capillary column from Phemomenex (Torrance, CA, USA) was used as stationary phase. Following oven temperature gradient was applied during the chromatographic run: maintained 70 °C for 2 min, increased at 150 °C at 25 °C/min rate, then increased at 200 °C at 12 °C/min rate and finally to 310 °C at 6 °C/min rate and maintained for 7 min. The injection volume was 1.2 µL, using a sandwich injection consisting of 0.3 µL of sample followed by 0.9 µL of syringe standard solution Lindane-D6. Pulse splitless injection was performed at an injector temperature of 230 °C. Mass spectrometer was operated in positive electron impact (EI) ionization, with source temperature set at 280 °C. For each analyte, the most abundant MRM (multiple reaction monitoring) transitions, optimized using the reference solutions of the analytes, were monitored. Monitored parent and fragment ions together with the fragmentation parameters are shown in Table S2.

### 2.6. Validation Parameters

LC-MS/MS and GC-MS/MS method validation was performed in accordance with the EU guideline SANTE/11312/2021 [35]. For the validation study, *Curcuma longa* (turmeric) and *Withania somnifera* extracts were used as representative matrices. Extracts were purchased from local market, ground, homogenized and stored at room temperature prior to the validation study. The following performance characteristics were evaluated during the validation study: linearity, sensitivity, specificity/selectivity, trueness, precision, and measurement uncertainty. Linearity was assessed using a five-point matrix-matched calibration curve. The deviation of the back-calculated concentrations from the nominal (true) concentrations was required to be within ±20%. Specificity and selectivity were evaluated by analyzing reagent blanks and matrix blank samples to verify the absence of interfering signals. Limits of quantification (LOQs) were established in accordance with the applicable legislation and were set at or below the MRLs specified in Regulation (EU) No 396/2005. For analytes subject to a complex residue definition, compliance with the LOQ requirement was verified by confirming that the sum of the LOQs of the individual residue components was equal to or lower than the corresponding MRL, in accordance with SANCO/12574/2014 [36]. Trueness and precision were determined by analyzing six replicate samples spiked at two concentration levels corresponding to the limit of quantification (LOQ) and 10× LOQ. Trueness was expressed as the mean recovery (%), while precision was expressed as the relative standard deviation (RSD) and coefficient of variation (CV%).

### 2.7. Real Samples Collection

Ten plant extract samples were collected in the local herbal stores. The samples comprised one *Garcinia cambogia* extract, two *Centella asiatica* (gotu kola) extracts, two *C. longa* extracts, one *Ginkgo biloba* extract, one *Berberis aristata* extract, one bromelain extract, one *Boswellia serrata* extract, and one food supplement in dosed form containing a mixture of plant extracts. All the samples were stored at room temperature prior to analysis.

### 2.8. Real Samples Preparation and Analysis

All samples were analyzed according to the analytical procedure described above. Sample preparation was common to both LC–MS/MS and GC–MS/MS analyses. Each analytical batch included the following quality control samples, processed together with the unknown samples: a reagent blank, a matrix blank, and a matrix sample fortified at LOQ (0.01 or 0.02 mg/kg, depending on the analyte). The presence of a suspect analyte was considered confirmed when the retention time matched that of the corresponding reference standard within ±0.1 min and the ion ratio complied with the acceptance criterion of ±30%. For quantitative analysis a four-point matrix-matched calibration curve was prepared from blank matrix extracts fortified after extraction. Calibration levels were 1.5, 10, 25, and 50 ng/mL for GC–MS/MS, and 1.5, 2.5, 10, and 60 ng/mL for LC–MS/MS. Quantification of the detected analytes was performed by interpolation from the corresponding matrix-matched calibration curve. For GC-MS/MS analysis, analyte concentrations were calculated by multiplying the interpolated concentration by the extraction solvent volume (10 mL) and the dilution factor (4, arising from the syringe dilution), and dividing by the exact sample weight. Similarly, for LC-MS/MS the concentrations of the analytes were obtained by multiplying the interpolated concentration by the extraction solvent volume (10 mL) and the dilution factor 10, arising from the syringe dilution, and dividing by the exact sample weight. Analytes were quantified against their corresponding IS, where applicable, or by direct calibration when no internal standard was used. Specifically, for GC-MS/MS analysis analytes: Aldrin, Bifenthrin, Biphenyl, Bromopropylate, Buprofezin, cis-Chlordane, oxy-Chlordane, trans-Chlordane, Coumaphos, Cyfluthrin, lambda-Cyhalothrin, Cypermethrin, Cyprodinil, Deltamethrin, Dieldrin, Diphenylamine, Endosulfan-alpha, Endosulfan-beta, Endrin, EPN, Etoxazole, Fenazaquin, Fenvalerate, Fipronil, Fludioxonil, tau-Fluvalinate, alpha-HCH, Heptachlor, Heptachlor-exo-epoxide, Heptachlor-endo-epoxide, Iprodione, gamma-HCH (Lindane), Mepanipyrim, Methoxychlor, Nitrofen, 2,4′-DDD, 2,4′-DDE, 2,4′-DDT, Oxadixyl, Pendimethalin, Permethrin, Pirimiphos ethyl, 4,4′-DDD, 4,4′-DDE, 4,4′-DDT, Pyridaben Pyriproxyfen, Quintozene, Tebufenpyrad, Tetradifon, Tetramethrin were quantified against IS alpha-HCH-D6, while HCB was quantified using the HCB-13C6. For LC-MS/MS analysis only the analyte Amitraz was quantified with IS Amitraz-D12.

## 3. Results and Discussion

### 3.1. Optimization of Sample Preparation

**Figure 2:**
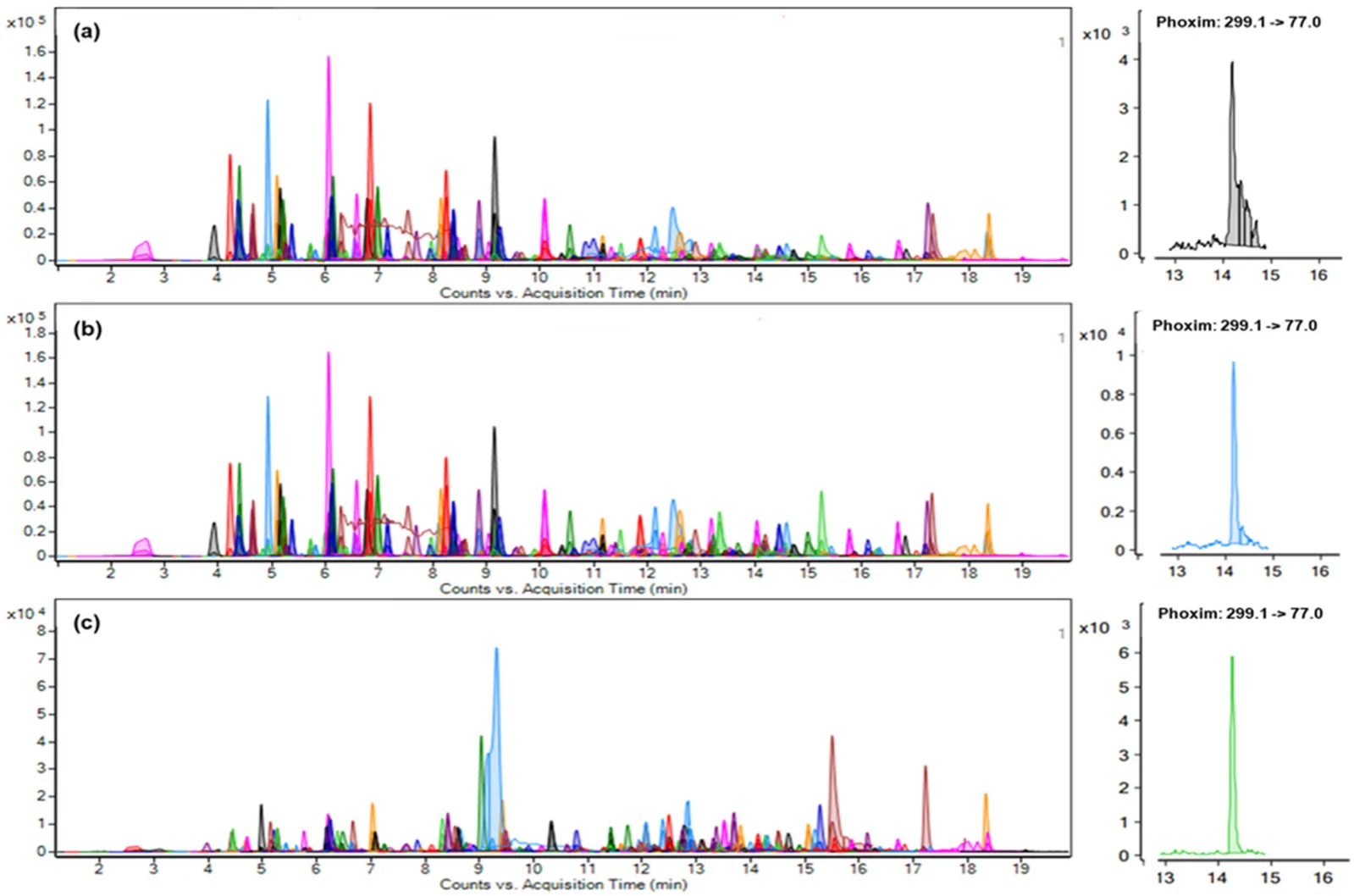
LC-MS/MS MRM chromatograms of turmeric samples subjected to three different clean-up procedures, with the selected Phoxim transition shown on the right as a representative example. (**a**) Turmeric sample fortified at 50 µg/kg (50 ppb) purified with 150 mg PSA and 900 mg MgSO_4_ (Cleanert MAS-Q). (**b**) Turmeric sample fortified at 50 µg/kg (50 ppb) treated with 450 mg PSA, 300 mg C18, 50 mg GCB, and 900 mg MgSO_4_ (DisQuE^TM^); (**c**) Turmeric sample fortified at 10 µg/kg (10 ppb) treated with 450 mg PSA, 300 mg C18, 50 mg GCB, and 900 mg MgSO_4_ (DisQuE^TM^), followed by pass-through OASIS^®^ WAX cartridge clean-up.

Extraction and purification steps are essential for removing interfering matrix components and enhancing the detection of the target analytes prior to instrumental analysis. The optimization of these procedures largely depends on the complexity of the sample matrix. Solid food supplements may contain dried plant materials, concentrated botanical extracts, and a variety of excipients added for technological or formulation purposes. This wide diversity in composition results in highly variable matrix characteristics, making the development of robust and universally applicable extraction and clean-up procedures particularly challenging. As dietary supplements in solid dosage forms are low-moisture matrices, a hydration step was incorporated prior to extraction to prevent excessive absorption of the extraction solvent (acetonitrile) by the dry matrix and to facilitate the formation of a homogeneous slurry, ensuring efficient extraction of the target analytes [18,19,37,38]. QuEChERS method with acetonitrile was used for the extraction of target analytes [21]. Following centrifugation, the extract was subjected to a freezing step to promote the precipitation and separation of co-extracted lipids and proteins prior to additional clean-up steps [18]. The clean-up steps were necessary to lower potential interfering co-extracts. Two dSPE clean-up procedures were evaluated using turmeric-based extract samples. The first procedure employed dSPE tubes Cleanert MAS-Q containing 900 mg MgSO_4_ and 150 mg PSA, while the second used DisQuE^TM^ tubes containing 900 mg MgSO_4_, 450 mg PSA, 300 mg C18, and 50 mg GCB. Overall, the clean-up procedure including C18 and GCB provided superior removal of matrix interferences, resulting in cleaner chromatograms. Figure 2 exemplifies the MRM chromatograms of turmeric samples prepared in different clean-up conditions. The improved performance is likely attributable to the enhanced adsorption of co-extracted matrix components, including pigments and lipids. Consequently, the dSPE composition containing PSA, C18, and GCB was selected for subsequent analyses. To further improve extract cleanliness and reduce matrix effects, an additional purification step using Oasis^®^ WAX cartridges was incorporated into the sample preparation procedure. This supplementary clean-up effectively removed residual matrix co-extractives, resulting in improved chromatographic performance and reduced analytical interferences for multiple analytes (Figure 2).

### 3.2. Method Validation

Given the complexity of botanical matrices, the pronounced matrix effects, and the substantial compositional variability of plant extracts, linearity was evaluated using a matrix-matched calibration curve. A blank matrix was extracted and purified following the extraction procedure and then reconstituted with pooled reference standard solutions immediately before instrumental analysis. For each analyte, a five-point matrix-matched calibration curve was prepared over the concentration range of 1.5–2.5–10–60–140 ng/mL for LC-MS/MS analysis and 1.5–2–10–25–50 ng/mL for GC-MS/MS analysis. All calibration curves showed a correlation coefficient (R^2^) ≥ 0.99, and the relative deviation between the back-calculated and nominal concentrations of the calibration standards did not exceed ±20%. Specificity and selectivity were assessed using reagent blanks and matrix blanks. No significant interfering signals were observed at the retention times of the analytes, and any interfering peaks did not exceed 30% of the peak area of the corresponding analyte at LOQ. The sensitivity of the method was assessed by determining the LOQ for each analyte. The LOQs were established in accordance with Regulation (EC) 396/2005 to ensure compliance with the applicable residue definitions and their corresponding MRLs. Following the guidance provided in SANCO/12574/2014, it was verified that the sum of the LOQs of the individual components was less than or equal to the relevant MRL, thereby demonstrating adequate method sensitivity. Where this criterion could not be met, it was demonstrated that sample compliance could be reliably assessed by taking measurement uncertainty into account. For LC-MS/MS detection the LOQs for all analytes was 0.01 mg/kg, while for GC-MS/MS detection the LOQs were 0.01 and 0.02 mg/kg.

Validation results for trueness and precision are shown in Tables S3 and S4.

For LC-MS/MS analysis, the mean recoveries (%) were in the range 70–120% and CV% values did not exceed 20% (Table S3) for 131 analytes for *W. somnifera* matrix. For 41 analytes the mean recoveries were outside the range 70–120% but stayed within 30–140% range [35]. The analytes Fenpyroximate and Metaflumizoneshowed the CV% of 22% and 21% at the LOQ spiking level, respectively (Table S3). For the *C. longa* matrix, 14 analytes showed mean recoveries within the extended range of 30–140%, with CV values below 20% for all of them. However, the CV exceeded 20% for Azinphos-ethyl, Bromuconazole, Flufenacet, Fluquinconazole, and Tetraconazole (Table S3). Although the precision criterion was not fully met for every analyte in both matrices, it was satisfied for each analyte in at least one of the two matrices considered. For analytes Isoxaflutole and Propiconazole, matrix interferences were observed at the LOQ level for *W. somnifera*, and for Profenofos in *C. longa* matrix, preventing the calculation of recoveries For the GC-MS/MS method, 48 analytes fulfilled the validation criteria, exhibiting mean recoveries between 70 and 120% and CV% values ≤ 20% for the matrix *W. somnifera* (Table S4). For 30 analytes in the *W. somnifera* matrix and 5 analytes in *C. longa* matrix, mean recoveries were outside the 70–120% range but remained within 30–140%. The analytes Biphenyl, Chlorobenzilate, Paraoxon methyl, Terbufos showed CV% values between 21 and 25% at the LOQ spiking level in *W. somnifera* matrix and analyte Isocarbofos exceeded target CV% with the value of 22%. Considering that a limited number of analytes were outside the established acceptance criteria for one of the validated matrices, the validation parameters were considered acceptable in view of the overall validation results conducted on two plant matrices. Nevertheless, ongoing method performance evaluation should be carried out during routine analysis, as recommended by the SANTE guidelines, to ensure the correct application of the method [35].

Measurement uncertainty was estimated using the bottom-up approach and expressed as relative expanded uncertainty. The uncertainty contributions associated with trueness, precision, and the calibration curve were evaluated individually for each analyte. For the remaining sources of uncertainty, including reference material purity, standard solution preparation, sample weighing, and dilution, a fixed contribution of 15% was applied. In accordance with SANTE/11312/2021, a default expanded uncertainty of 50% may be applied for official control purposes. The uncertainty estimation demonstrated that the relative expanded uncertainty for the validated analytes ranged from 17.7% to 32.3%. Therefore, the default expanded uncertainty of 50% was considered applicable.

**Figure 3:**
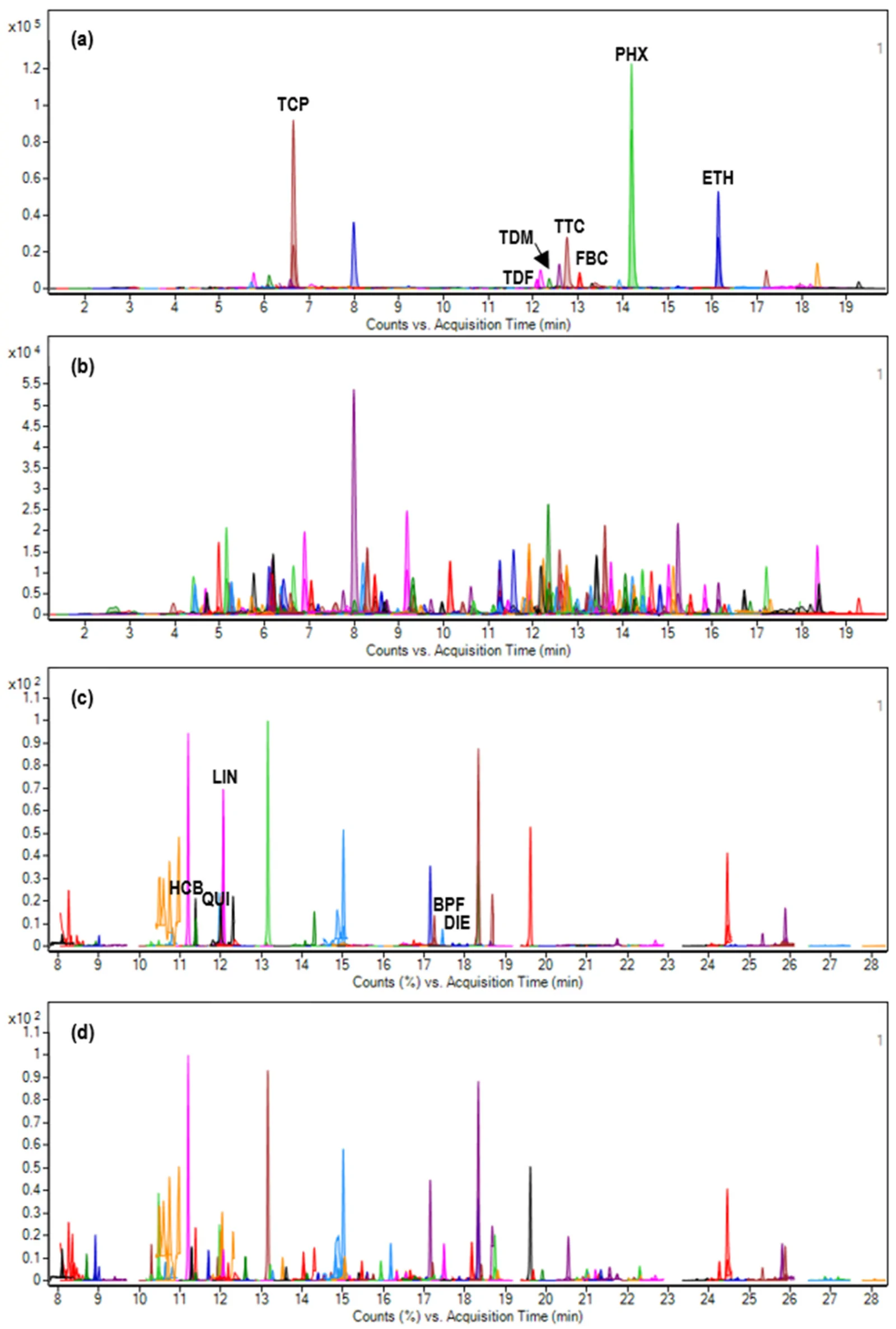
LC-MS/MS (**a**,**b**) and GC-MS/MS (**c**,**d**) MRM chromatograms of proficiency test sample (**a**,**c**) and control blank ginseng sample fortified at the concentration of 10 µg/kg (**b**,**d**). TCP: Thiacloprid, TDF: Triadimefon, TDM: Triadimenol, TTC: Triticonazole, FBC: Fenbuconazole, PHX: Phoxim, ETH: Ethion, QUI: Quintozene, LIN: Lindane, BPF: Buprofezin, DIE: Dieldrin.

As an additional assessment of the performance of the developed LC–MS/MS and GC–MS/MS methods, the laboratory participated in a FAPAS^®^ proficiency test for pesticide residues in herbal medicine, using ginseng as the test matrix. The proficiency-testing sample was treated and analyzed under routine analytical conditions using the developed methods (Figure 3).

The results were evaluated against the assigned values provided by the proficiency-testing provider and are reported in Table 1. The obtained z-scores ranged from −1.4 to +0.4, with all results falling within the predefined acceptance criterion (|z| ≤ 2). No false-positive or false-negative results were observed. These findings provide additional independent support for the suitability of the developed methods for the multiresidue determination of pesticides in botanicals.

### 3.3. Real Samples Analysis

The validated LC-MS/MS and GC-MS/MS methods were applied to 10 botanical samples to demonstrate their applicability to plant extracts/food supplements. Multiple pesticide residues were detected in three samples: one *Centella asiatica* extract, one *Boswellia serrata* extract, and one sample containing a mixture of plant extracts (Table 2). Sample S2 contained seven different pesticide residues at concentrations above the respective LOQs, including insecticides cypermethrin, fipronil (reported as sum of fipronil and its metabolite fipronil sulfone) acetamiprid, and fungicides dimethomorph, pyraclostrobin, tebuconazole, and tricyclazole. Three of the detected compounds are not approved for use in the EU. The *Boswellia serrata* extract contained one non-approved fungicide and two insecticides, while the sample containing a mixture of plant extracts was contaminated with two insecticides that are not approved for use in the EU. Overall, the concentrations of the detected pesticide residues ranged from 0.01 to 0.19 mg/kg. Current legislation does not establish MRLs specifically for finished food supplements; however, a useful point of comparison can be provided by the MRLs established for herbs and spices. For the sum of fipronil and its metabolite fipronil sulfone, the MRL established for various spices is 0.005 mg/kg (which represents the lowest limit for the analytical determination). Accordingly, the concentration detected in the *Centella asiatica* sample exceeded this value by at least a factor of 20. For acetamiprid, the MRLs established for spices range from 0.05 to 0.1 mg/kg, depending on the plant species and the plant part used. The concentration detected in the same sample (0.19 mg/kg) was therefore approximately two to four times higher than these reference values. It should be emphasized that direct comparison with MRLs established for herbs and spices does not constitute a regulatory compliance assessment for food supplements, as these limits apply to different commodities and are intended to reflect specific agricultural and dietary exposure scenarios. Nevertheless, such comparisons can provide a useful indication of the magnitude of the residues detected. The occurrence of multiple pesticide residues in botanical products has also been reported in previous studies. Hayward et al. detected between one and twelve pesticide residues per botanical dietary supplement, with ginseng-containing products showing the highest number of residues; concentrations of individual pesticides reached up to 2.8 mg/kg [20]. Similarly, Palenikova et al. reported the presence of one to fourteen pesticide residues in dietary supplements analyzed using combined GC-MS/MS and LC-HRMS approaches, with ginseng-containing products again exhibiting the highest degree of contamination. The highest concentration reported was 92.7 µg/kg of butralin in a ginseng- and guarana-containing sample [23]. Fenclova et al. detected one to three pesticide residues in milk thistle-based dietary supplements. Notably, one sample contained malathion, chlorpyrifos, and carbendazim, with concentrations of individual residues reaching up to 118 µg/kg [39]. The application of the validated methods to real samples obtained from the local market yielded results consistent with previously published reports. Importantly, the growing body of evidence demonstrating the occurrence of multiple pesticide residues in botanical products strengthens the rationale for conducting cumulative risk assessments of pesticide exposure from food supplements. Such assessments are particularly relevant given the broad consumer population of these products, which includes young people, adults of reproductive age, and older individuals. Considering the potential for simultaneous exposure to multiple pesticide residues, continued monitoring and comprehensive risk assessment are warranted to better characterize the potential risks associated with the consumption of contaminated food supplements.

## 4. Conclusions

In the present study, LC-MS/MS and GC-MS/MS methods for the quantification of pesticide residues in botanical products were successfully validated in accordance with current regulatory requirements. Both methods employed a common sample preparation procedure expanding the range of detectable target analytes while maintaining efficient sample preparation workflow for routine analysis. The satisfactory validation performance, together with the results obtained in an independent proficiency-testing experiment, support the potential applicability of the developed method for reliable multiresidue screening and quantitative determination in complex botanical matrices. Nevertheless, botanical matrices continue to pose significant challenges for broad-spectrum multi-residue pesticide analysis. Validation studies conducted on a limited number of representative matrices have highlighted the difficulty of meeting the stringent validation criteria established by current guidelines for all analytes across different matrices, underscoring the strong dependence of method performance on matrix composition. These findings highlight the need to harmonize validation approaches, including the selection and number of matrices to be considered, and to investigate whether the use of pooled matrices could provide a suitable compromise between analytical reliability and practical feasibility. Such strategies could help ensure the robustness and applicability of validated methods while ensuring that their implementation in routine laboratories remains feasible in terms of workload and resource requirements. Furthermore, standard addition method may be required as a complementary approach to obtain reliable pesticide residue concentrations following multi-residue quantification when matrix effects cannot be adequately compensated, as suggested by SANTE guidelines [35]. Its application is performed on a compound- and matrix-specific basis, implying the additional time and resources required for routine implementation, especially than multiple contaminations are detected. The validated methods were subsequently applied to ten botanical plant extract samples purchased on the local market. Multiple pesticide residues were detected in three samples, with one sample containing seven different residues, three of which are not approved for use in the EU. Despite the limited number of samples analyzed, these findings further highlight the importance of continued monitoring to assess the quality and safety of botanical products.

## Figures and Tables

**Table 1:** Results of the analysis of Fapas^®^ proficiency testing.

Matrix	Pesticide Residue	Method	Assigned Value (µg/kg)	z-Score
Ginseng	Ethion	LC-MS/MS	76.8	−0.3
	Fenbuconazole		79.2	−0.3
	Phoxim		169	−0.1
	Thiacloprid		75.7	−0.5
	Triadimefon		75.6	−0.3
	Triadimenol		66.3	−0.5
	Triticonazole		144	−0.9
	Buprofezin	GC-MS/MS	122	0.0
	Dieldrin		88.0	−1.2
	HCB		69.0	0.4
	Lindane		96.0	−0.1
	Quintozene		107	−1.4

**Table 2:** Pesticide residues detected in 10 samples analysed with validated LC- and GC-MS/MS methods.

Sample	Plant Extract	Detected Analyte	Method	[mg/kg]	Approved(A)/ Not Approved (B) in EU *
S1	*Garcinia cambogia*	<LOQ			
S2	*Centella asiatica*	Cypermethrin	GC-MS/MS	0.065	A
		Fipronil + Fipronil sulfone	GC-MS/MS	0.1	B
		Acetamiprid	LC-MS/MS	0.19	A
		Dimethomorph	LC-MS/MS	0.01	B
		Pyraclostrobin	LC-MS/MS	0.11	A
		Tebuconazole	LC-MS/MS	0.02	A
		Tricyclazole	LC-MS/MS	0.02	B
S3	*Curcuma longa*	<LOQ			
S4	Plant mix	Chlorpyriphos ethyl	LC-MS/MS	0.038	B
		Propoxur	LC-MS/MS	0.019	B
S5	*Ginkgo biloba*	<LOQ			
S6	*Berberis aristata*	<LOQ			
S7	*Centella asiatica*	<LOQ			
S8	*Curcuma longa*	<LOQ			
S9	Bromelain	<LOQ			
S10	*Boswellia serrata*	Buprofezin	GC-MS/MS	0.081	A
		Fenazaquin	GC-MS/MS	0.013	A
		Propiconazole	LC-MS/MS	0.013	B

LOQ: limit of quantification. * Current status for active substance under Reg. (EC) No. 1107/2009 (as per https://food.ec.europa.eu/plants/pesticides/eu-pesticides-database_en, accessed on 14 September 2026).

## Data Availability

The original contributions presented in the study are included in the article/Supplementary Material, further inquiries can be directed to the corresponding author.

## Supplementary Materials

The following supporting information can be downloaded at https://www.mdpi.com/article/10.3390/foods15213892/s1, Table S1: MS/MS parameters for target analytes and corresponding ISs used for LC-MS/MS analysis; Table S2: MS/MS parameters for target analytes and corresponding ISs used for GC-MS/MS analysis; Table S3: Trueness and precision expressed as mean recovery and relative standard deviation (RSD) and coefficient of variation (CV), respectively, for each analyte in LC-MS/MS method determined at 2 spiking levels in two plant matrices; Table S4: Trueness and precision expressed as mean recovery and relative standard deviation (RSD) and coefficient of variation (CV), respectively, for each analyte in GC-MS/MS method determined at 2 spiking levels in two plant matrices.
