# Supplementary material for: Validation of Multi-Residue GC-MS/MS and LC-MS/MS Methods for the Analysis of Pesticide Residues in Botanicals
Source: Foods. 2026 Oct 30;15(21):3892. doi: 10.3390/foods15213892 (PMC13763230; doi:10.3390/foods15213892)
Supplement: Supplementary file 1 [file foods-15-03892-s001.zip › foods-4600327-supplementary.pdf]

# - Supplementary materials -

***

Validation of Multi-Residue GC-MS/MS and LC-MS/MS Methods for the Analysis of Pesticide Residues in Botanicals

Elena Butovskaya [1+], Giuseppina Castrezzati [1], Giampiero Migliorato [1], Gregorio Peron [2] and Mara Gasparini [1]

[1]Food and Feed Chemistry Department, Istituto Zooprofilattico Sperimentale della Lombardia e dell'Emilia Romagna "Bruno Ubertini" (IZSLER), 25124 Brescia, Italy;

[2]Department of Molecular and Translational Medicine, University of Brescia, 25123 Brescia, Italy;

***

Table S1 MS/MS parameters for target analytes and ISs used for LC-MS/MS analysis. (*Quantifier ion)

Analyte	Precursor ion (m/z)	Product ion (m/z)	Fragmentor (V)	Collision energy (eV)
Acephate	184	94.9, 142.9 *	60	24, 4
Acetamiprid	223.1	90, 126 *	108	36, 20
Aldicarb	208, 116	115.8, 88.9 *	80	10, 4
Aldicarb-sulfone	223	76, 86*	80	4, 12
Aldicarb-sulfoxide	207.1	89, 132 *	64	8, 0
Ametoctradin	276.2	149, 176*	200	44, 44
Amitraz	294.2	122,163*	70	32, 12
Azinphos-ethyl	346	77, 131.9*	50	50, 12
Azinphos-methyl	318	77, 131.9*	86	50, 12
Azoxystrobin	404.1	344.1, 372.1*	116	24, 8
Benzovindiflupyr	398.1	286.1, 342.1*	140	32, 16
Bitertanol	338.2	70.1*, 99.1	86	4, 12
Bixafen	414	265.8, 393.8*	111	24, 12
Boscalid	343	140, 307.1*	75	20, 20
Bromuconazole	375.9	69.9, 158.8*	116	24, 36
Bupirimate	317.2	108, 166.1*	145	28, 24
Carbaryl	202.1	127, 145*	50	28, 4
Carbendazim	192.1	132, 159.9*	91	36, 16
Carbofuran	222.1	123*, 165	88	20, 4
Carbofuran 3-hydroxy	238.1	107.1, 163.1*	84	32, 8
Chlorantraniliprole	482	283.7*, 450.7	96	12, 16
Chlorbufam	241	154, 172*	75	15, 10
Chlorfenvinphos	359	99, 155*	90	36, 8
Chlorpropham	214.1	125.9, 171.9*	55	28, 4
Chlorpyriphos ethyl	350	96.9*, 197.9	100	40, 16
Clofentezine	303	102, 138*	90	40, 12
Clothianidin	250	131.9, 169*	90	16, 8
Cyazofamid	325.1	107.9*, 260.8	80	16, 8
Cyflufenamid	413.1	241, 295*	120	24, 12
Cyflumetofen	465.2	172.9*, 249	125	24, 8
Cyhalofop-butyl	375	120, 256*	70	32, 12
Cymoxanil	199.1	111, 128*	70	20, 8
Cyproconazole	292.1	70.1*, 125	126	16, 36

Analyte	Precursor ion (m/z)	Product ion (m/z)	Fragmentor (V)	Collision energy (eV)
Demeton-S-methyl	231	60.9, 88.9*	50	40, 10
Demeton-S-methyl-sulfone	263	109, 169*	120	28, 12
Demeton-S-methyl-sulfoxide	247	105, 109*	90	8, 28
Diazinon	305.1	153.1, 169*	140	20, 20
Dichlorvos	221	78.9, 108.9*	106	28, 16
Diethofencarb	268.1	124, 226.1*	84	32, 4
Difenoconazole	406.1	251*, 337	138	24, 12
Diflubenzuron	311.04	141, 158*	105	36, 12
Diflufenican	395	246, 266*	135	35, 20
Dimethoate	230	124.9, 198.9*	80	20, 4
Dimethomorph	388.1	165.1, 301.1*	165	32, 20
Diniconazole	326.1	69.9*, 158.8	126	32, 36
Dinotefuran	203	129, 157*	90	8, 4
Epoxiconazole	330.1	101, 121*	116	50, 24
Ethiofencarb	226.1	107*, 164	80	5, 5
Ethion	385	96.9, 199*	85	48, 4
Ethoprophos	243.1	96.9, 130.9*	80	36, 20
Etofenprox	394.2	107, 177*	100	48, 12
Famoxadone	392.2	238, 331.1*	90	12, 4
Fenamidone	312.1	92.1*, 236.1	115	24, 12
Fenamiphos	304.1	202, 217*	140	36, 20
Fenamiphos-sulfone	336.1	266*, 308	135	16, 12
Fenamiphos-sulfoxide	320.1	108, 233*	120	44, 24
Fenbuconazole	337.1	70*, 125	70	16, 44
Fenoxycarb	302.1	88*, 116	108	16, 4
Fenpropathrin	367.2	97.1, 125.1*	85	40, 16
Fenpropidin	274.3	86, 146.9*	127	32, 32
Fenpropimorph	304.3	130, 147.1*	132	28, 32
Fenpyrazamine	332.15	216.1, 230.1*	125	28, 16
Fenpyroximate	422.2	107.1*, 366.1	147	50, 36
Fensulfothion	309	156.9, 280.8*	106	28, 12
Fenthion	279	168.9*, 246.9	106	16, 12
Fenthion-oxon	263.1	216, 231*	138	24, 12
Fenthion-oxon-sulfone	295	104.1, 217.1*	145	24, 16
Fenthion-oxon-sulfoxide	279.1	104, 264*	133	28, 16
Fenthion-sulfone	311	109, 125*	140	28, 20
Fenthion-sulfoxide	295	109, 280*	140	36, 16
Flonicamid	230.1	174, 203*	127	20, 16
Flubendiamide	683	273.9, 407.9*	86	36, 4
Flufenacet	364.1	151.9*, 193.9	86	20, 8
Flufenoxuron	489.1	141, 158*	120	50, 16
Fluopicolide	383	144.8, 172.8*	101	50, 24
Fluopyram	397.1	173*, 207.9	127	32, 20
Flupyradifurone	289, 291	126*, 128	150	30, 30
Fluquinconazole	376	307*, 349	70	28, 20
Flusilazole	316.1	165, 247.1*	140	28, 16
Flutolanil	324.1	242, 262*	125	28, 16
Flutriafol	302.1	70*, 123	116	16, 28
Fluxapyroxad	382.1	342.1, 362*	125	20, 12
Formetanate	222.1	119.9, 164.9*	96	28, 16
Fosthiazate	284.1	104*, 228	90	24, 4
Furathiocarb	383.2	195*, 252.1	112	12, 8
Hexaconazole	314.1	70*, 159	118	20, 40
Hexythiazox	353.1	168.1, 228*	115	24, 12
Imazalil	297.1	69, 158.9*	121	20, 24

Analyte	Precursor ion (m/z)	Product ion (m/z)	Fragmentor (V)	Collision energy (eV)
Imidacloprid	256.1	175.1*, 209.1	102	20, 12
Indoxacarb	528.1	150*, 203	118	20, 45
Iprovalicarb	321.2	119.1*, 203.1	100	20, 4
Isofetamid	360.2	124.9*, 210	95	32, 4
Isoprothiolane	291.1	188.8, 230.8*	75	20, 8
Isoxaflutole	360.1	219.8, 250.8*	111	44, 12
Kresoxim-methyl	314.1	115.9, 206*	80	12, 5
Linuron	249	159.9*, 182	70	16, 12
Lufenuron	511	140.8, 157.8*	116	50, 20
Malaoxon	315.1	99, 127*	90	20, 8
Malathion	331	126.9*, 284.7	86	8, 4
Mandipropamid	412.1	125, 328.1*	120	44, 12
Mefenpyr-diethyl	373.1	159.8, 326.8*	91	36, 8
Mefentrifluconazole	398.1	70.1*, 182	135	24, 32
Metaflumizone	507	178*, 287	125	25, 25
Metalaxyl-M	280.2	192.1, 220.1*	109	16, 8
Metamitron	203.1	77.1, 175.1*	130	32, 16
Metconazole	320.2	70.1*, 125	70	24, 48
Methamidophos	142	94*, 125	80	12, 8
Methidathion	303	85, 145*	70	16, 4
Methiocarb	226.1	121.1*, 169.1	60	15, 0
Methomyl	163	88, 106*	55	20, 5
Methoxyfenozide	369.2	149*, 313.1	85	12, 4
Metobromuron	259	148, 169.9*	105	12, 16
Metrafenone	409.1	208.9*, 226.7	101	12, 20
Metribuzin	215.1	83.9, 186.9*	111	24, 16
Monocrotophos	224.1	127, 193*	60	12, 4
Myclobutanil	289.1	70*, 125	125	16, 36
N-(2,4-Dimethylphenyl)formamide	150.1	77, 132*	120	50, 12
N-(2,4-Dimethylphenyl)-N`-methylformamidine	163	107, 122*	106	28, 16
Omethoate	214	125*, 183	90	20, 4
Oxadiargyl	341.05	151, 223*	160	28, 12
Oxamyl	237.1	72.1*, 90	52	15, 20
Paclobutrazol	294.1	70.1*, 125	75	16, 44
Parathion	291.9	94, 235.9*	85	40, 12
Penconazole	284.1	70.1*, 159	108	12, 28
Pencycuron	329.1	125*, 218	130	36, 12
Penflufen	318.2	141, 234*	105	36, 16
Penthiopyrad	360.14	177, 276*	130	36, 12
Phosalone	368	111, 181.9*	100	48, 8
Phosphamidon	300	127, 174*	84	8, 8
Phoxim	299.1	77*, 129	90	36, 8
Pirimicarb	239.1	72*, 182.1	108	20, 12
Pirimicarb-desmethyl	225.2	72.1*, 168.1	110	15, 10
Pirimiphos-methyl	306.1	108, 164.1*	145	32, 20
Prochloraz	376	70.1, 308*	96	28, 8
Profenofos	373	128, 302.8*	130	48, 16
Propamocarb	189.2	74, 102*	108	24, 12
Propargite	368	174.9, 230.9*	86	16, 8
Propham	180.1	119.9, 138*	55	16, 4
Propiconazole	342.1	69.1, 159*	144	16, 28
Propoxur	210.1	93, 111*	74	24, 8
Prosulfocarb	252.1	91*, 128.1	105	24, 8
Prothioconazole-desthio	312.1	70*, 125	85	20, 44

Analyte	Precursor ion (m/z)	Product ion (m/z)	Fragmentor (V)	Collision energy (eV)
Prothiofos	345	268.9, 240.9 *	105	8, 16
Pyraclostrobin	388.1	163, 194*	110	24, 8
Pyrazophos	374.1	194, 222.1*	135	36, 20
Pyrimethanil	200.1	82, 107*	148	28, 24
Quinalphos	299	147, 162.9*	115	20, 24
Quinoclamine	208	77.1*, 105	140	44, 28
Spinetoram J	748.5	98, 142*	190	50, 32
Spinetoram L	760	98, 142*	190	35, 35
Spinosyn-A	732.5	98.1, 142.1*	80	45, 32
Spinosyn-D	746.5	98, 142.1*	80	50, 15
Spirodiclofen	411.1	71.1*, 312.9	81	15, 5
Spiroxamine	374.2	216*, 302.1	140	36, 16
Sulfoxaflor	278.1	154, 174*	75	32, 4
Tebuconazole	308.2	70*, 125	126	24, 48
Tebufenozide	353.2	133*, 297.1	60	20, 4
Terbuthylazine	230	96, 174*	120	20, 20
Tetraconazole	372	70, 159*	144	20, 36
Thiacloprid	253	90.1, 126*	108	40, 20
Thiametoxam	292	181, 211.1*	86	20, 8
Thiodicarb	355.1	88*, 108	84	12, 8
Thiophanate-methyl	343.1	151*, 311	105	20, 5
Tolclofos-methyl	301	124.9*, 174.9	106	12, 28
Tolfenpyrad	384.15	154, 197.1*	180	50, 28
Triadimefon	294.1	69*, 197	116	20, 12
Triadimenol	296.1	69.9*, 98.9	75	5, 10
Triazophos	314.1	119, 162*	125	36, 16
Tricyclazole	190.1	136, 163*	160	32, 24
Trifloxystrobin	409.1	145, 186*	116	50, 16
Triflumizole	346.1	73, 278*	100	12, 4
Triflumizole-metabolite-FM-6-1	295.1	73.1*, 215	115	16, 24
Triflumuron	359	139, 156*	121	40, 16
Triticonazole	318.1	70*, 125	126	16, 40
Zoxamide	336	159, 187*	120	44, 20
Amitraz-D12	306.3	169.2	95	12
Azinphos-ethyl-D10	356.1	132	77	12
Carbendazim-D3	195.1	160	116	16
Cyromazine-D4	171.1	85	96	20
Dimethoate-D6	236	204.9	70	4
Etofenprox-D5	399	182	91	12
Imidacloprid-D4	260.1	213.1	104	12
Propiconazole-D5	347.1	159	155	36

***

Table S2 MS/MS parameters for target analytes and corresponding ISs used for GC-MS/MS analysis. (*Quantifier ion)

Analyte	Precursor ion (m/z)	Product ion (m/z)	Collision energy (eV)
Aclonifen	264, 212	194*, 182	30, 30
Aldrin	262.9, 254.9	192.9 *, 220	35, 20
Bifenthrin	181	166 *, 154	15, 10
Biphenyl	154, 153	115 *, 127	30, 25
Bromopropylate	338.8, 185	182.9 *, 157	20, 15
Buprofezin	190, 172	175 *, 115	5, 15
Cadusafos	159, 158.7	131, 97 *	5, 20
cis-Chlordane	372.9, 271.9	265.9, 236.9 *	20, 15
oxy-Chlordane	184.9, 114.9	121, 51.1*	15, 25
trans-Chlordane	372.9, 271.7	265.9, 236.9 *	20, 15
Chlorfenapyr	246.9, 136.9	227, 102 *	20, 20
Chlorobenzilate	251, 139	139 *, 111	15, 10
Chlorpyrifos methyl	286	270.9, 93 *	20, 25
Coumaphos	361.9	109.2, 81 *	15, 40
Cyfluthrin	226, 163	206, 127 *	15, 5
lambda-Cyhalothrin	197	161, 141 *	10, 10
Cypermethrin	164.9, 163	91, 127 *	10, 5
Cyprodinil	224	208 *, 118	20, 40
2,4′-DDD	237, 235	165.2 *, 199	20, 20
2,4′-DDE	316, 246	246, 176 *	15, 30
2,4′-DDT	235	199, 165.2 *	15, 20
4,4′-DDD	235	199, 165 *	20, 20
4,4′-DDE	316, 246	246, 176 *	15, 30
4,4′-DDT	235	199, 165.2 *	20, 20
Deltamethrin	253	174, 93 *	5, 20
Dieldrin	277, 262.9	241 *, 193	15, 35
Diphenylamine	169, 168	168 *, 139.1	15, 40
Disulfoton	153, 88	96.9, 60 *	10, 5
Disulfoton-sulfone	213, 124.9	96.9, 96.9 *	15, 10
Endosulfan-alpha	241, 206.9	206, 172 *	15, 15
Endosulfan-beta	240, 206.9	205, 172 *	10, 15
Endosulfan sulfate	387, 271.9	289, 236.9 *	5, 20
Endrin	262.8, 244.8	193 *, 173	35, 40
EPN	169, 157	141.1 *, 110	5, 12
Etoxazole	299.9, 204	269.9, 176.1 *	20, 10
Fenazaquin	160	145 *, 117	5, 20
Fenitrothion	277	260, 109 *	5, 25
Fenpropathrin	265, 181	210, 152 *	20, 20
Fenvalerate	167, 125	125 *, 89.1	10, 25
Fipronil	367, 350.8	213 *, 254.8	40, 15
Fipronil-sulfone	383	255 *, 241	25, 15
Fludioxonil	248	154, 127 *	25, 30
tau-Fluvalinate	250	200, 55 *	20, 20
Hexachlorobenzene	283.8	248.8 *, 213.9	15, 30
alpha-HCH	219, 181	183, 145 *	10, 10
beta-HCH	219, 181	183, 145 *	10, 10
gamma-HCH (Lindane)	219, 181	183, 145 *	10, 10
Heptachlor	273.7, 271.7	238.9, 236.9 *	15, 15
Heptachlor-exo-epoxide	352.8, 216.9	262.9 *, 181.9	15, 25
Heptachlor-endo-epoxide	216.9, 182.9	181.9, 118.9 *	25, 25
Iprodione	314	245, 56 *	10, 20
Isocarbophos	135.9, 120	108 *, 92	15, 10
Isofenphos-methyl	199, 198.4	93 *, 121.1	30, 5
Mepanipyrim	222.2, 222	207.2, 220 *	15, 25
Methacrifos	207.9	180 *, 93	5, 15
Methoxychlor	227	169, 141.1 *	15, 40

Analyte	Precursor ion (m/z)	Product ion (m/z)	Collision energy (eV)
Mevinphos	192, 127	127, 109 *	10, 10
Nitrofen	282.9, 202	161.9, 139.1 *	20, 20
Oxadixyl	163	132 *, 117	15, 25
Paraoxon-methyl	230	136, 106 *	5, 18
Parathion-methyl	263	109.1 *, 79.1	15, 35
Pendimethalin	252.1	208 *, 161.2	5, 10
Permethrin	163	127 *, 91	5, 10
Phenthoate	274	246, 125 *	5, 15
Phosmet	160	105, 77 *	15, 25
Pirimiphos ethyl	318.1	182, 166.1 *	10, 10
Procymidone	283	96 *, 67	10, 40
Propyzamide	173	145 *, 109	10, 25
Pyridaben	147	132 *, 117	10, 20
Pyriproxyfen	136	96, 78 *	20, 20
Quintozene	295, 237	237 *, 119	14, 24
Tebufenpyrad	333, 275.9	171, 171.1 *	15, 10
Tefluthrin	197	161, 141 *	5, 10
Terbufos	230.9	175, 129 *	10, 20
Tetradifon	356, 229	159 *, 201	10, 25
Tetramethrin	164	107 *, 77	10, 25
Trifluralin	306, 264	264 *, 160	5, 15
Vinclozolin	212, 197.9	172, 145 *	25, 20
alfa-HCH-D6	224	187	20
HCB-13C6	289.8	254.8	30
4,4′-DDE-D8	324	254	20
2,4′-DDT-D8	243	173	20
4,4′-DDT-D8	243.1	173.1	20
Chlorpyrifos-methyl-D6	292	99	20
Lindane-D6	224	185	10

***

Table S3 Trueness and precision expressed as mean recovery and relative standard deviation (RSD) and coefficient of variation (CV), respectively, for each analyte in LC-MS/MS method determined at 2 spiking levels in two plant matrices.

Analyte	*Withania somnifera*						*Curcuma longa*					
	Spiking level (LOQ = 0.01 mg/kg)			Spiking level (0.1 mg/kg)			Spiking level (LOQ = 0.01 mg/kg)			Spiking level (0.1 mg/kg)		
	Mean recovery %	RSD	CV (%)	Mean recovery %	RSD	CV (%)	Mean recovery %	RSD	CV (%)	Mean recovery %	RSD	CV (%)
Acephate	81	3,6	4	59	2,7	5	100	3,0	3	82	2,8	3
Acetamiprid	97	4,3	4	87	0,8	1	108	0,8	1	95	3,0	3
Aldicarb	107	2,9	3	83	2,0	2	113	5,1	5	91	2,7	3
Aldicarb-sulfone	112	10,5	9	83	2,5	3	111	12,7	11	93	4,6	5
Aldicarb-sulfoxide	94	2,1	2	80	1,7	2	105	3,9	4	88	2,4	3
Ametoctradin	68	3,5	5	44	1,1	3	90	3,4	4	61	2,6	4
Amitraz	122	8	6	42	1	3	130	2,1	2	111	3,2	3
Azinphos-ethyl	89	7,0	8	76	2,5	3	120	22,3	18	80	27,3	34
Azinphos-methyl	89	4,4	5	73	1,5	2	114	5,1	4	84	4,4	5
Azoxystrobin	104	6,3	6	86	1,8	2	109	2,5	2	89	2,6	3
Benzovindiflupyr	96	7,1	7	77	2,6	3	95	11,9	13	90	6,3	7
Bitertanol	86	4,7	5	62	6,0	10	91	15,4	17	79	2,3	3
Bixafen	90	9,3	10	73	5,3	7	98	18,8	19	87	2,8	3
Boscalid	106	14,4	14	65	5,6	9	99	9,9	10	75	7,5	10
Bromuconazole	89	13,7	15	79	5,0	6	82	19,2	23	70	5,8	8
Bupirimate	103	4,6	4	81	2,2	3	100	6,5	6	95	3,4	4
Carbaryl	92	2,6	3	74	2,0	3	107	4,9	5	87	2,5	3
Carbendazim	112	3,3	3	75	1,7	2	113	6,2	5	72	2,2	3
Carbofuran	105	2,3	2	91	1,5	2	112	3,9	3	95	2,5	3
Carbofuran-3-hydroxy	97	7,4	8	75	2,5	3	110	9,2	8	94	3,8	4
Chlorantraniliprole	49	7,4	15	30	4,2	14	67	7,4	11	49	6,8	14
Chlorbufam	99	9,1	9	59	4,2	7	95	12,5	13	79	10,9	14
Chlorfenvinphos	101	8,0	8	87	1,6	2	108	5,3	5	94	3,7	4
Chlorpropham	90	9,2	10	63	4,7	7	108	12,8	12	79	10,5	13
Chlorpyriphos-ethyl	99	8,8	9	72	1,3	2	111	11,4	10	80	11,4	14
Clofentezine	62	3,3	5	37	1,9	5	96	6,7	7	75	2,5	3
Clothianidin	93	5,5	6	71	2,3	3	109	8,3	8	85	3,2	4
Cyazofamid	103	5,2	5	91	3,6	4	105	6,8	6	83	3,6	4
Cyflufenamid	105	4,2	4	91	2,5	3	118	7,0	6	94	2,6	3
Cyflumetofen	104	5,0	5	79	2,3	3	100	6,2	6	90	7,9	9
Cyhalofop-butyl	105	15,6	15	82	3,8	5	113	7,6	7	86	1,9	2

Cymoxanil	108	8,0	7	80	2,7	3	105	4,3	4	91	1,4	2
Cyproconazole	91	4,6	5	78	4,4	6	106	9,3	9	80	5,4	7
Demeton-S-methyl	104	0,8	1	89	1,5	2	106	1,6	2	93	2,0	2
Demeton-S-methyl-sulfone	103	3,3	3	88	1,3	1	114	5,6	5	95	2,6	3
Demeton-S-methyl-sulfoxide	99	2,7	3	81	1,3	2	102	1,3	1	85	2,0	2
Diazinon	102	2,5	2	84	2,6	3	105	4,5	4	90	2,9	3
Dichlorvos	103	4,3	4	85	1,8	2	106	9,4	9	89	2,4	3
Diethofencarb	101	2,1	2	78	0,6	1	110	5,1	5	91	2,6	3
Difenoconazole	94	6,0	6	73	2,4	3	108	7,1	7	75	3,5	5
Diflubenzuron	59	3,4	6	36	3,7	10	62	4,9	8	55	5,6	10
Diflufenican	68	4,9	7	43	2,8	6	108	8,7	8	83	2,7	3
Dimethoate	98	2,2	2	87	1,2	1	112	2,5	2	98	2,5	3
Dimethomorph	96	4,9	5	78	2,1	3	111	4,7	4	84	6,1	7
Diniconazole	79	5,8	7	52	3,7	7	74	12,0	16	70	2,7	4
Dinotefuran	94	2,6	3	81	2,3	3	103	4,0	4	90	2,6	3
Epoxiconazole	97	6,9	7	81	1,9	2	110	12,2	11	83	3,4	4
Ethiofencarb	97	3,2	3	82	1,4	2	110	2,9	3	91	2,1	2
Ethion	103	7,0	7	87	2,2	3	112	5,1	5	87	6,1	7
Ethoprophos	99	2,0	2	87	1,6	2	107	4,0	4	85	11,3	13
Etofenprox	82	3,0	4	58	2,5	4	101	3,0	3	78	2,0	3
Famoxadone	101	9,5	9	80	4,5	6	113	15,7	14	90	5,7	6
Fenamidone	93	2,9	3	75	1,4	2	112	5,4	5	90	3,5	4
Fenamiphos	93	4,4	5	78	2,4	3	104	5,8	6	88	2,3	3
Fenamiphos-sulfone	105	3,7	3	88	2,6	3	108	4,6	4	86	1,6	2
Fenamiphos-sulfoxide	103	2,6	3	84	2,2	3	103	1,6	2	81	1,5	2
Fenbuconazole	98	4,0	4	80	2,6	3	107	9,7	9	81	3,4	4
Fenoxycarb	94	5,9	6	73	2,0	3	109	9,6	9	88	3,1	4
Fenpropathrin	99	5,5	6	77	3,0	4	111	4,3	4	87	0,9	1
Fenpropidin	87	3,8	4	48	1,8	4	73	1,5	2	47	4,7	10
Fenpropimorph	94	2,7	3	69	2,3	3	84	5,9	7	75	1,7	2
Fenpyrazamine	97	5,8	6	84	1,7	2	108	4,4	4	87	3,1	4
Fenpyroximate	74	16,6	22	56	1,5	3	107	4,2	4	81	3,0	4
Fensulfothion	107	5,1	5	87	2,2	2	113	4,6	4	87	2,2	3
Fenthion	89	8,8	10	76	2,5	3	104	7,7	7	86	4,2	5
Fenthion-oxon	100	2,6	3	84	0,8	1	109	2,2	2	89	1,8	2
Fenthion-oxon-sulfone	107	1,0	1	91	2,3	3	107	5,1	5	94	2,7	3
Fenthion-oxon-sulfoxide	95	4,0	4	85	0,7	1	105	3,3	3	90	2,3	3
Fenthion-sulfone	114	6,7	6	88	1,0	1	112	4,4	4	91	3,1	3
Fenthion-sulfoxide	102	2,8	3	86	0,9	1	110	5,5	5	89	3,3	4

Flonicamid	111	7,4	7	84	2,3	3	116	11,3	10	96	4,0	4
Flubendiamide	109	19,3	18	89	5,4	6	122	15,7	13	91	9,1	10
Flufenacet	103	5,4	5	92	4,0	4	124	10,3	8	86	18,8	22
Flufenoxuron	72	4,2	6	55	2,7	5	105	6,1	6	86	2,1	2
Fluopicolide	104	4,6	4	88	2,3	3	111	6,8	6	86	11,9	14
Fluopyram	100	5,8	6	87	2,0	2	107	6,8	6	89	8,6	10
Flupyradifurone	96	8,3	9	83	2,5	3	89	5,8	7	95	3,1	3
Fluquinconazole	84	14,2	17	76	7,4	10	107	16,2	15	74	16,4	22
Flusilazole	112	8,8	8	83	4,2	5	116	2,3	2	86	1,5	2
Flutolanil	101	6,1	6	85	2,1	3	97	7,1	7	89	11,1	12
Flutriafol	99	1,8	2	79	4,8	6	103	2,1	2	85	3,9	5
Fluxapyroxad	100	5,2	5	89	3,6	4	114	10,9	10	90	13,0	14
Formetanate	105	0,6	1	76	0,3	0	109	1,9	2	80	1,3	2
Fosthiazate	105	1,8	2	89	1,4	2	107	2,9	3	92	1,9	2
Furathiocarb	104	3,5	3	84	2,9	3	117	2,3	2	89	1,9	2
Hexaconazole	95	7,2	8	69	5,5	8	98	5,2	5	74	3,4	5
Hexythiazox	88	4,8	5	66	2,9	4	97	5,6	6	74	5,2	7
Imazalil	95	7,6	8	73	2,0	3	99	5,8	6	66	1,8	3
Imidacloprid	105	3,5	3	80	1,9	2	103	9,7	9	86	3,5	4
Indoxacarb	116	15,9	14	91	5,5	6	101	16,8	17	86	5,2	6
Iprovalicarb	106	5,9	6	87	1,8	2	115	8,7	8	88	11,8	13
Isofetamid	98	8,8	9	91	1,3	1	97	8,0	8	87	11,8	14
Isoprothiolane	102	5,5	5	87	1,9	2	113	5,9	5	91	6,1	7
Isoxaflutole	interf			107	19,7	18	88	5,7	6	73	12,5	17
Kresoxim-methyl	100	8,5	9	80	1,9	2	113	8,9	8	90	3,8	4
Linuron	93	4,0	4	68	1,8	3	110	4,8	4	88	6,1	7
Lufenuron	73	9,3	13	51	2,2	4	106	13,6	13	85	8,8	10
Malaoxon	106	2,4	2	90	1,2	1	108	2,5	2	93	1,5	2
Malathion	105	5,3	5	89	2,7	3	117	9,0	8	93	7,8	8
Mandipropamid	101	7,0	7	78	1,1	1	113	4,3	4	91	2,6	3
Mefenpyr-diethyl	106	5,7	5	91	2,9	3	117	5,6	5	91	3,0	3
Mefentrifluconazole	96	5,2	5	78	6,2	8	95	14,4	15	83	4,1	5
Metaflumizone	66	13,6	21	31	2,3	7	96	19,7	20	87	8,0	9
Metalaxyl-M	104	1,6	2	88	1,6	2	110	2,7	2	91	2,4	3
Metamitron	86	8,6	10	78	2,1	3	95	12,6	13	87	1,8	2
Metconazole	94	8,0	8	68	4,4	6	96	7,0	7	78	2,6	3
Methamidophos	85	0,6	1	66	1,6	2	96	1,9	2	77	1,6	2
Methidathion	102	4,4	4	84	2,5	3	107	2,9	3	87	3,5	4
Methiocarb	96	3,4	4	77	1,9	2	114	6,2	5	85	5,4	6

Methomyl	104	2,0	2	88	1,6	2	112	4,0	4	94	1,1	1
Methoxyfenozide	99	5,9	6	84	2,4	3	106	8,3	8	89	6,5	7
Metobromuron	95	5,1	5	84	3,7	4	65	2,7	4	86	4,8	6
Metrafenone	96	4,7	5	75	1,8	2	108	5,2	5	88	2,4	3
Metribuzin	65	5,8	9	89	3,0	3	116	7,8	7	91	3,3	4
Monocrotophos	99	10,0	10	88	3,2	4	112	4,5	4	92	1,9	2
Myclobutanil	112	5,7	5	91	4,3	5	98	9,9	10	81	13,5	17
N-(2,4-Dimethylphenyl)formamide	101	4,2	4	84	3,5	4	106	5,5	5	89	2,6	3
N-(2,4-Dimethylphenyl)-N`-methylformamidine	103	1,6	2	57	1,4	2	94	2,9	3	61	3,5	6
Omethoate	91	4,0	4	77	2,0	3	104	2,0	2	87	2,6	3
Oxadiargyl	100	11,5	12	98	6,2	6	90	13,2	15	90	9,7	11
Oxamyl	101	1,4	1	83	1,3	2	108	2,5	2	90	1,9	2
Paclobutrazol	101	5,6	6	78	4,4	6	111	2,6	2	86	6,0	7
Parathion	81	9,2	11	87	5,3	6	105	19,4	19	94	11,2	12
Penconazole	106	6,1	6	76	1,5	2	104	8,0	8	82	4,8	6
Pencycuron	85	3,3	4	81	2,0	3	99	4,2	4	80	2,7	3
Penflufen	98	5,4	6	87	1,3	1	93	2,8	3	92	4,5	5
Penthiopyrad	98	3,8	4	93	1,8	2	100	3,9	4	95	2,0	2
Phosalone	96	9,0	9	73	2,4	3	102	11,3	11	85	4,6	5
Phosphamidon	105	2,9	3	87	1,3	1	109	6,1	6	89	1,9	2
Phoxim	106	7,1	7	86	2,6	3	111	4,0	4	94	2,5	3
Pirimicarb	95	0,9	1	79	0,9	1	108	3,0	3	89	1,6	2
Pirimicarb-desmethyl	94	1,9	2	78	1,7	2	105	2,1	2	87	1,7	2
Pirimiphos-methyl	98	4,2	4	81	1,4	2	107	4,5	4	89	2,6	3
Prochloraz	90	4,6	5	75	1,9	2	92	4,5	5	73	1,7	2
Profenofos	93	3,1	3	75	2,2	3	interf			83	8,6	10
Propamocarb	82	1,0	1	60	2,0	3	68	2,1	3	45	2,8	6
Propargite	99	7,9	8	82	1,6	2	108	2,0	2	87	1,9	2
Propham	100	1,6	2	77	3,0	4	111	7,7	7	87	2,4	3
Propiconazole	interf			78	4,9	6	101	13,0	13	77	2,1	3
Propoxur	103	2,7	3	90	1,5	2	111	2,3	2	94	2,0	2
Prosulfocarb	98	3,7	4	78	1,9	2	106	2,7	3	85	1,7	2
Prothioconazole-desthio	83	3,6	4	69	3,1	5	100	8,4	8	76	3,2	4
Prothiofos	86	3,9	5	59	1,2	2	104	5,7	6	77	2,5	3
Pyraclostrobin	68	4,3	6	50	2,2	4	110	1,7	2	88	1,8	2
Pyrazophos	61	5,3	9	45	2,9	6	107	7,5	7	86	1,8	2
Pyrimethanil	71	4,1	6	42	1,3	3	87	4,8	6	73	4,4	6
Quinalphos	99	4,8	5	72	1,4	2	119	6,6	6	90	4,1	5

Quinoclamine	63	7,3	12	46	1,8	4	97	6,9	7	78	4,9	6
Spinetoram	86	3,1	4	53	2,9	5	85	4,7	5	55	3,2	6
Spinosyn-A	93	1,6	2	59	2,4	4	98	2,8	3	61	2,7	4
Spinosyn-D	87	11,1	13	56	2,5	4	83	9,6	11	54	3,4	6
Spirodiclofen	119	3,1	3	90	5,4	6	107	4,9	5	81	1,9	2
Spiroxamine	85	2,4	3	57	2,2	4	77	1,7	2	47	2,9	6
Sulfoxaflor	111	3,3	3	91	1,9	2	112	3,6	3	97	2,3	2
Tebuconazole	87	4,4	5	70	3,1	4	109	7,0	6	86	2,5	3
Tebufenozide	101	4,4	4	84	1,7	2	111	4,7	4	89	3,0	3
Terbuthylazine	94	2,0	2	77	2,5	3	111	5,5	5	84	8,3	10
Tetraconazole	113	14,3	13	91	5,5	6	117	24,0	21	85	25,3	30
Thiacloprid	96	3,1	3	83	1,2	1	98	2,2	2	87	1,9	2
Thiametoxam	100	4,2	4	85	1,5	2	106	7,5	7	93	3,5	4
Thiodicarb	83	2,8	3	65	1,7	3	107	2,5	2	89	2,2	3
Thiophanate-methyl	63	2,7	4	55	2,9	5	69	2,6	4	66	6,3	10
Tolclofos-methyl	102	9,2	9	79	3,0	4	117	13,5	12	83	4,6	5
Tolfenpyrad	72	7,6	10	55	1,8	3	98	4,3	4	76	2,6	4
Triadimefon	106	11,3	11	87	2,9	3	108	10,1	9	80	15,4	19
Triadimenol	110	13,1	12	79	4,8	6	113	10,2	9	84	5,8	7
Triazophos	94	4,8	5	82	1,1	1	112	5,0	4	89	4,4	5
Tricyclazole	74	3,4	5	58	1,3	2	84	1,7	2	67	1,8	3
Trifloxystrobin	104	5,5	5	86	2,0	2	114	4,2	4	92	1,3	1
Triflumizole	96	3,6	4	78	1,3	2	90	3,1	3	82	1,6	2
Triflumizole-metabolite-FM-6-1	98	2,8	3	80	1,5	2	92	4,3	5	88	2,1	2
Triflumuron	83	7,1	9	65	4,5	7	108	7,2	7	88	3,8	4
Triticonazole	88	4,7	5	74	7,1	10	88	7,4	8	74	6,5	9
Zoxamide	91	6,8	7	82	3,5	4	105	5,5	5	87	3,5	4

***

Table S3 Trueness and precision expressed as mean recovery and relative standard deviation (RSD) and coefficient of variation (CV), respectively, for each analyte in GC-MS/MS method determined at 2 spiking levels in two plant matrices.

Analyte	LOQ (mg/kg)	*Withania somnifera*						*Curcuma longa*					
		Spiking level (LOQ)			Spiking level (0.1 mg/kg)			Spiking level (LOQ)			Spiking level (0.1 mg/kg)		
		Mean recovery %	RSD	CV (%)	Mean recovery %	RSD	CV (%)	Mean recovery %	RSD	CV (%)	Mean recovery %	RSD	CV (%)
Aclonifen	0,02	55	4,2	8	60	9,2	15	63	5,1	8	76	3,6	5
Aldrin	0,01	69	5,3	8	61	2,6	4	92	5,7	6	94	4,0	4
Bifenthrin	0,01	96	9,9	10	74	3,4	5	109	3,7	3	110	2,8	3
Biphenyl	0,01	52	11,1	21	46	4,5	10	114	11,2	10	88	4,5	5
Bromopropylate	0,01	77	8,5	11	59	5,0	8	92	5,2	6	101	7,4	7
Buprofezin	0,02	104	10,1	10	78	2,6	3	81	6,5	8	135	10,8	8
Cadusafos	0,01	87	4,4	5	81	3,9	5	77	3,1	4	76	2,6	3
cis-Chlordane	0,01	81	3,3	4	72	3,1	4	103	11,2	11	105	4,9	5
oxy-Chlordane	0,01	84	3,5	4	73	2,0	3	102	5,3	5	109	3,5	3
trans-Chlordane	0,01	76	3,7	5	72	2,1	3	104	6,3	6	104	3,6	3
Chlorfenapyr	0,02	105	6,5	6	78	4,1	5	74	5,7	8	81	3,8	5
Chlorobenzilate	0,01	78	19,5	25	86	7,0	8	71	4,7	7	96	4,3	4
Chlorpyrifos-methyl	0,01	83	9,7	12	65	6,3	10	74	4,3	6	72	3,0	4
Coumaphos	0,01	59	4,9	8	32	3,6	11	81	5,3	7	93	6,8	7
Cyfluthrin	0,01	88	10,9	12	68	2,3	3	100	9,7	10	105	4,8	5
lambda-Cyhalothrin	0,01	94	11,4	12	72	3,6	5	87	3,2	4	74	2,5	3
Cypermethrin	0,01	102	6,2	6	69	4,4	6	103	8,3	8	109	4,0	4
Cyprodinil	0,01	47	8,3	18	30	5,4	18	71	7,7	11	90	5,1	6
2,4′-DDD	0,01	70	6,2	9	62	4,1	7	97	4,2	4	104	4,5	4
2,4′-DDE	0,01	54	6,1	11	49	4,5	9	81	3,9	5	88	4,0	4
2,4′-DDT	0,01	59	8,9	15	47	4,3	9	77	6,6	9	86	3,8	4
4,4′-DDD	0,01	70	5,3	8	60	4,7	8	101	3,8	4	102	4,6	4
4,4′-DDE	0,01	48	3,8	8	55	5,3	10	95	10,5	11	95	5,1	5
4,4′-DDT	0,01	64	9,2	14	45	5,0	11	79	7,8	10	87	4,1	5
Deltamethrin	0,01	86	12,8	15	65	4,0	6	102	8,1	8	105	3,5	3
Dieldrin	0,01	86	8,6	10	73	2,9	4	99	11,0	11	108	6,0	6
Diphenylamine	0,01	58	10,9	19	52	5,4	10	102	11,0	11	97	4,1	4
Disulfoton	0,01	84	11,3	14	75	3,5	5	81	5,6	7	76	3,1	4
Disulfoton-sulfone	0,01	106	12,5	12	85	4,7	6	95	17,6	19	76	2,4	3
Endosulfan-alpha	0,01	87	6,1	7	75	3,6	5	105	7,9	8	113	4,2	4
Endosulfan-beta	0,01	93	5,1	5	79	3,9	5	113	6,2	5	110	1,8	2
Endosulfan-sulfate	0,01	91	6,3	7	80	3,9	5	72	4,2	6	75	2,3	3

Endrin	0,02	85	10,8	13	68	4,5	7	78	5,3	7	101	5,8	6
EPN	0,01	87	13,7	16	70	4,2	6	109	2,6	2	105	5,3	5
Etoxazole	0,01	98	9,8	10	78	3,5	4	109	9,5	9	110	6,7	6
Fenazaquin	0,01	52	7,0	13	34	3,6	10	75	4,9	7	81	2,9	4
Fenitrothion	0,01	100	14,3	14	80	4,8	6	76	6,6	9	77	3,7	5
Fenpropathrin	0,01	82	16,5	20	73	4,1	6	86	8,6	10	83	4,3	5
Fenvalerate/Esfenvalerate	0,01	97	8,9	9	70	3,0	4	110	3,3	3	110	2,6	2
Fipronil	0,01	110	5,4	5	91	5,1	6	101	4,1	4	112	7,1	6
Fipronil sulfone	0,01	100	11,4	11	89	5,3	6	77	9,4	12	85	7,0	8
Fludioxonil	0,01	73	12,6	17	54	6,1	11	95	8,8	9	109	4,9	4
Fluvalinate-tau	0,01	99	9,0	9	69	4,3	6	99	4,3	4	110	5,3	5
Hexachlorobenzene	0,01	86	4,4	5	77	3,0	4	113	4,5	4	113	4,9	4
alfa-HCH	0,01	86	3,2	4	78	2,4	3	114	2,4	2	114	2,7	2
beta-HCH	0,01	81	13,4	17	74	2,6	4	79	4,5	6	77	2,6	3
gamma-HCH (Lindane)	0,01	87	2,8	3	79	2,8	4	98	9,9	10	113	2,6	2
Heptachlor	0,01	84	4,0	5	72	2,3	3	101	5,4	5	105	3,8	4
Heptachlor-exo-epoxide	0,01	90	3,7	4	78	2,7	3	104	11,9	11	107	5,7	5
Heptachlor-endo-epoxide	0,02	92	4,1	4	76	2,8	4	88	12,3	14	115	7,8	7
Iprodione	0,02	105	10,5	10	86	7,6	9	85	10,2	12	109	4,2	4
Isocarbofos	0,02	84	4,9	6	86	4,3	5	52	11,5	22	83	5,7	7
Isofenphos-methyl	0,01	98	14,7	15	83	5,4	7	80	6,3	8	82	3,8	5
Mepanipyrim	0,01	46	5,2	11	30	4,1	13	96	7,8	8	104	4,6	4
Methacrifos	0,01	93	13,1	14	76	6,1	8	87	3,3	4	81	2,3	3
Methoxychlor	0,01	94	13,9	15	64	4,4	7	97	13,4	14	108	7,3	7
Mevinphos	0,02	108	3,3	3	79	7,8	10	60	7,9	13	133	15,3	12
Nitrofen	0,01	68	7,8	12	57	5,1	9	85	5,4	6	93	4,3	5
Oxadixyl	0,01	106	13,9	13	85	4,2	5	82	4,2	5	79	3,1	4
Paraoxon-methyl	0,01	95	20,5	22	82	7,3	9	85	9,8	12	74	5,1	7
Parathion-methyl	0,01	98	13,8	14	78	5,6	7	78	5,0	6	78	3,8	5
Pendimethalin	0,01	87	11,8	13	75	6,3	8	102	4,5	4	113	4,8	4
Permethrin	0,01	86	13,7	16	63	3,1	5	105	6,6	6	107	2,9	3
Phenthoate	0,01	97	13,3	14	80	5,6	7	100	2,4	2	84	6,6	8
Phosmet	0,01	99	12,0	12	61	5,1	8	80	6,6	8	68	3,7	5
Pirimiphos-ethyl	0,01	100	6,4	6	82	3,4	4	104	6,6	6	114	3,5	3
Procymidone	0,01	92	14,0	15	81	3,5	4	81	6,2	8	79	3,1	4
Propyzamide	0,02	87	4,3	5	77	3,0	4	67	3,0	4	81	9,0	11
Pyridaben	0,01	74	11,8	16	52	2,4	5	93	10,6	11	99	4,7	5
Pyriproxyfen	0,01	69	5,8	8	49	3,6	7	86	7,7	9	95	3,9	4
Quintozene	0,01	63	4,8	8	72	5,9	8	77	7,3	10	111	2,9	3

Tebufenpyrad	0,01	99	8,0	8	73	3,9	5	104	5,1	5	105	4,6	4
Tefluthrin	0,01	92	15,6	17	81	3,4	4	90	10,9	12	78	2,2	3
Terbufos	0,01	88	20,5	23	78	3,8	5	80	3,2	4	77	2,5	3
Tetradifon	0,01	72	5,4	7	56	3,7	7	71	5,5	8	87	5,5	6
Tetramethrin	0,01	104	12,5	12	83	3,8	5	107	6,4	6	109	5,8	5
Trifluralin	0,01	91	16,0	18	83	4,5	5	76	4,1	5	77	4,1	5
Vinclozolin	0,01	93	12,6	14	81	4,5	6	83	4,9	6	79	2,1	3
